# *Syn*ergy: A Web Resource for Exploring Gene Regulation in *Synechocystis* sp. PCC6803

**DOI:** 10.1371/journal.pone.0113496

**Published:** 2014-11-24

**Authors:** Niklas Mähler, Otilia Cheregi, Christiane Funk, Sergiu Netotea, Torgeir R. Hvidsten

**Affiliations:** 1 Department of Chemistry, Biotechnology and Food Science, Norwegian University of Life Sciences, Ås, Norway; 2 Department of Chemistry, Umeå University, Umeå, Sweden; 3 Umeå Plant Science Centre, Department of Plant Physiology, Umeå University, Umeå, Sweden; 4 Computational Life Science Cluster, Umeå University, Umeå, Sweden; National Institutes of Health, United States of America

## Abstract

Despite being a highly studied model organism, most genes of the cyanobacterium *Synechocystis* sp. PCC 6803 encode proteins with completely unknown function. To facilitate studies of gene regulation in *Synechocystis*, we have developed *Syn*ergy (http://synergy.plantgenie.org), a web application integrating co-expression networks and regulatory motif analysis. Co-expression networks were inferred from publicly available microarray experiments, while regulatory motifs were identified using a phylogenetic footprinting approach. Automatically discovered motifs were shown to be enriched in the network neighborhoods of regulatory proteins much more often than in the neighborhoods of non-regulatory genes, showing that the data provide a sound starting point for studying gene regulation in *Synechocystis*. Concordantly, we provide several case studies demonstrating that *Syn*ergy can be used to find biologically relevant regulatory mechanisms in *Synechocystis*. *Syn*ergy can be used to interactively perform analyses such as gene/motif search, network visualization and motif/function enrichment. Considering the importance of *Synechocystis* for photosynthesis and biofuel research, we believe that *Syn*ergy will become a valuable resource to the research community.

## Introduction

Cyanobacteria are the only prokaryotic organisms that produce oxygen in the process of photosynthesis, and are the ancestors of higher plant chloroplasts. Not only did cyanobacteria establish the aerobic Earth's atmosphere, they also play a crucial role in the global biochemical cycle today by fixing CO_2_ and producing half of the global biomass. Being prokaryotes, cyanobacteria can be genetically modified easily and due to their fast photoautotrophic growth, they have a great potential for large scale production of renewable biofuels [1], [2] and other valuable products [1], [3], [4]. The popularity of the cyanobacteria phylum in photosynthesis and biotechnology research is reflected in the high number of sequenced cyanobacterial genomes available in Cyanobase (http://genome.microbedb.jp/cyanobase/) [5] and other public databases [6]. After the genome of the unicellular fresh water cyanobacterium *Synechocystis* sp. PCC 6803 (hereafter *Synechocystis*) was sequenced in 1996 [7], large amounts of gene expression data have been generated from cells exposed to diverse experimental conditions. Identifying groups of genes with similar expression patterns (i.e. co-expressed genes) in such data sets allows inference of functional and regulatory similarities among genes. For example, light response in *Synechocystis* has been studied using gene co-expression networks [8]–[10]. While these studies give insight into how cells react to single modifications, only the integration of multiple transcriptome data sets will allow a holistic understanding of the cellular response. The first meta-analysis of transcriptomics data in *Synechocystis* used a co-expression network inferred from 163 different environmental and genetic perturbations to identify a large number of genes (referred to as the Core Transcriptional Response) that are commonly regulated under most perturbations [9]. The growing interest in integrated transcriptome analysis has also led to the development of a web database, CyanoEXpress [11]. Although this tool comprises a vast set of experimental data, and integrates microarray data obtained with different experimental platforms, its use is restricted to the visualization and analysis of gene expression clusters. However, genes regulated by the same transcription factor (i.e. co-regulated genes) should not only be co-expressed, but also contain similar *cis*-regulatory elements in their promoter region. In *Synechocystis*, co-expression has not yet been linked with motif discovery in order to obtain a more mechanistic understanding of gene regulation.

We have developed *Syn*ergy, a web resource for exploring *Synechocystis* gene regulation, which integrates co-expression network analysis with motif analysis. *Syn*ergy is available at http://synergy.plantgenie.org. Considering the importance of *Synechocystis* as a model organism in biofuel production [2] and photosynthetic research [12], [13], we believe *Synergy* will become a valuable resource to many researchers.

## Results and Discussion

**Figure 1:**
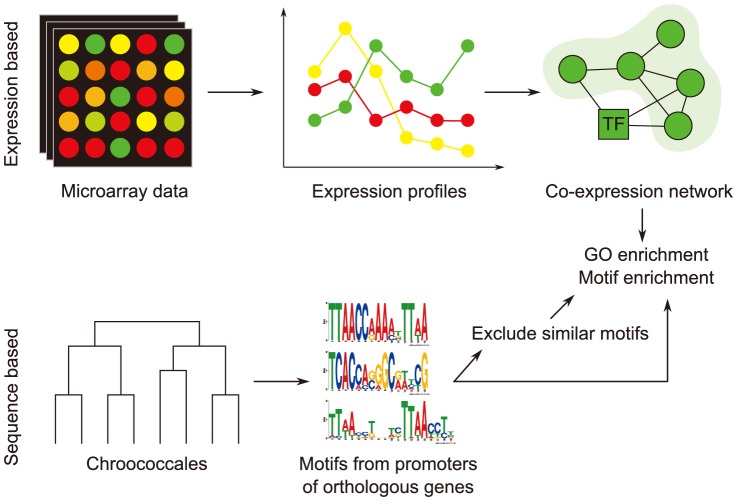
Overview of the data and methods used in the study. A co-expression network was inferred from gene expression, and promoter motifs were identified *de novo* from the genome sequences of orthologous species. The motif information was used to investigate if transcription factor neighborhoods were enriched for motifs compared to random network neighborhoods.

In this article we provide an integrated analysis of co-expression networks, promoter motifs and existing gene function annotations in *Synechocystis*. See Figure 1 for an overview.

### Co-expression network inference

Co-expression networks were inferred from 371 individual microarray experiments obtained from KEGG Expression (Table 1; http://www.genome.jp/kegg/expression/; [14]). We used locally corrected mutual information scores (CLR scores, see Materials and Methods) to measure co-expression between pairs of genes, and constructed co-expression networks by linking genes with a CLR score above a preset threshold. Thus, a co-expression network is a set of nodes representing genes, which are connected by links representing co-expression above a threshold. Since some of the expression values were missing in the published data, we decided to investigate their impact by inferring two different networks; one based on a subset of samples that contained expression values for all the genes across all microarrays (subset co-expression), and another one based on all microarrays (complete co-expression). The subset co-expression network contained 3,077 genes (i.e. nodes) and 59,595 links with a CLR score above 4.0, while the corresponding complete co-expression network contained 3,067 nodes and 52,081 links.

**Table 1:** References to the microarrays used in this study.

Reference	Arrays	Conditions
[38]	18	3
[39]	20	4
[40]	4	1
[41]	22	2
[42]	11	3
[43]	46	11
[44]	144	12
[45]	38	10
[46]	4	1
[47]	4	1
[48]	14	4
[49]	28	14
[50]	18	9
Total	371	

All data can be found at http://www.genome.jp/kegg/expression/.

**Figure 2:**
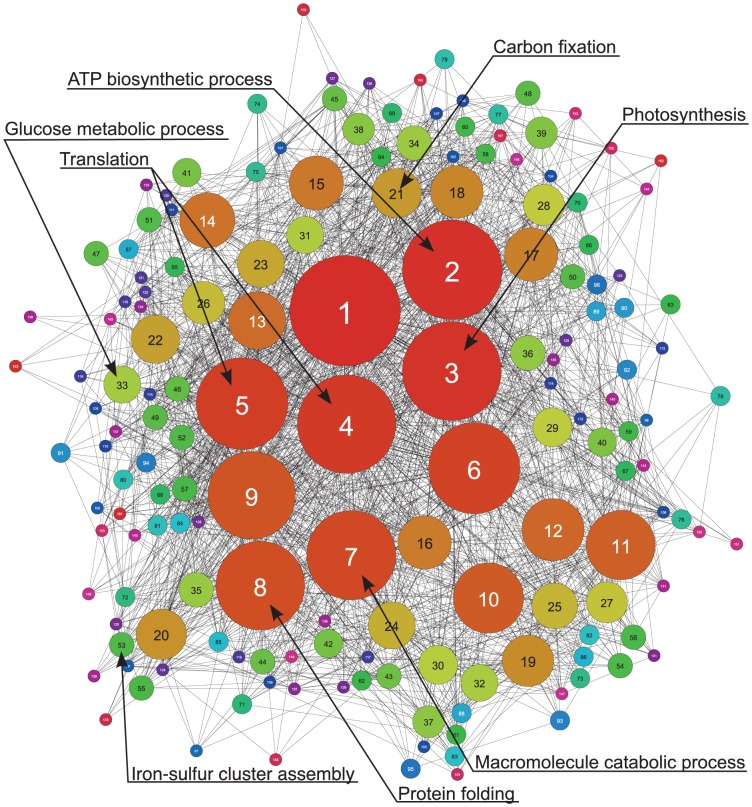
Clustered co-expression network. A clustered co-expression network derived from the complete co-expression network at a CLR threshold of 4.0. Each node corresponds to a set of clustered genes. The size of the nodes is proportional to the number of genes in the cluster. Two clusters are linked if they share at least one co-expressed gene pair. The annotations correspond to the most significantly enriched GO terms in the clusters (*q*<0.05).

Figure 2 shows a simplified version of the complete co-expression network where highly connected sub-networks are collapsed into single nodes (clusters) that thus represent several co-expressed genes (see Materials and Methods). Some of these clusters are associated with Gene Ontology (GO) [15] terms that are assigned more often to genes in that cluster than what one would expect by chance (false discovery rate (FDR) [16] corrected *p*-value <0.05 or, equivalently, *q*-value <0.05). We will refer to such statistically significant overrepresentation as *enrichment*. The dominating clusters in the network display genes encoding proteins related to energy metabolism, photosynthesis, translation and protein folding. These clusters stand out not only because they contain genes with stringent regulation under the majority of stress conditions tested, but also because these genes encode proteins with inter-functional dependency. As also previously noticed [9], the expression of ribosomal genes is correlated with the expression of energy producing pathways (photosynthesis and energy metabolism); shutting down the major energy producing pathways will result in temporary translational stop. Protection from reactive oxygen species (ROS) is of tremendous importance for an oxygen-producing organism like *Synechocystis*, which is reflected by the central location of the cluster representing genes coding for enzymes involved in protein folding.

Co-expression networks can be used to quantify the importance of a gene by reporting several different measures of *network centrality* calculated for the node representing that gene. The *degree centrality* of a node is defined as the fraction of all nodes in the network that are directly connected to it (i.e. neighbors). The *betweenness centrality* of a node is the fraction of times that node is in the shortest path between two other nodes in the network (the shortest path between two nodes in a network is the fewest number of links needed to travel from one node to the other). The 40 genes with the highest *degree*- and *betweenness- centrality* (average centrality of 0.179 and 0.008, respectively) in the complete co-expression network were both enriched for genes encoding proteins involved in the photosynthetic processes (GO:0015979: *photosynthesis*, *q*<0.001 and *q*<0.05, respectively). The complete results are available in file S1. The central role of these *photosynthesis* related genes within the gene regulation of *Synechocystis* is also supported by the relatively central location of its gene cluster (Cluster 3) in Figure 2. Functional enrichment of co-expression in the model plant *Arabidopsis thaliana* has also found a cluster of genes encoding proteins involved in *photosynthesis* in a central position [17]. This confirms the high conservation of *photosynthesis* related genes; in particular the regulation of these genes is highly conserved.

### Phylogenetic footprinting

**Figure 3:**
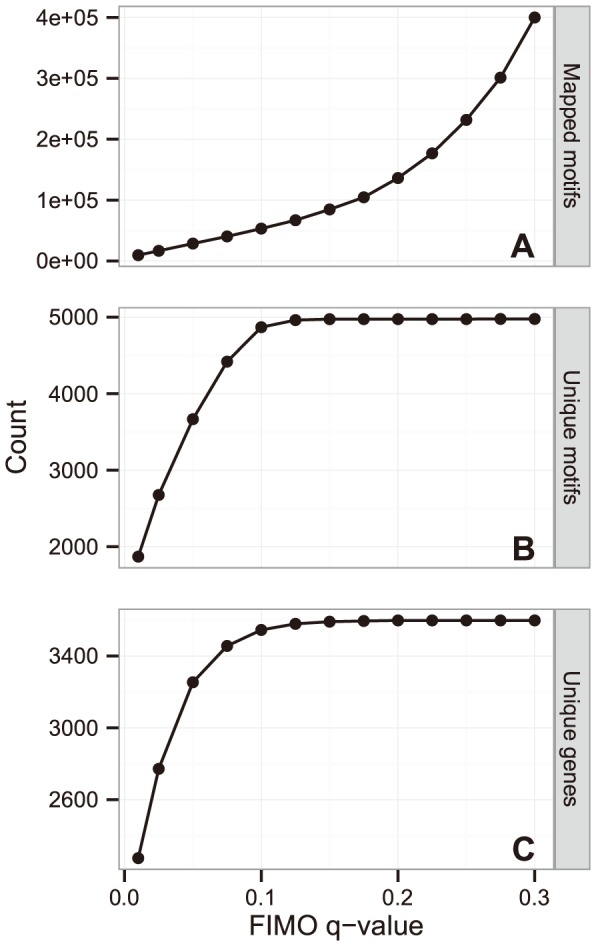
Central motifs mapped to *Synechocystis* promoters. The plots show the total number of times the central motifs were mapped to promoters (A), the number of unique motifs that were mapped (B) and the number of unique genes the motifs were mapped to (C) for different FIMO *q*-value thresholds.

Transcription factors (TFs) bind to regulatory elements in the promoter region of genes or operons to enhance or repress their transcription. Phylogenetic footprinting was used to identify conserved DNA motifs within promoters of orthologous genes, which would indicate functional regulatory elements. We identified 8,961 groups of orthologous genes in 22 Chroococcales genomes (see file S2 for a list of organisms) and searched for conserved DNA promoter motifs using *de novo* motif finding (see Materials and Methods). Since motifs were discovered from each group of orthologous genes independently, the resulting motif set contained as many as 15,306 motifs that could be mapped to *Synechocystis* promoters, of which many were very similar or even identical. To obtain a more representative motif set, we inferred a *motif similarity network*, identified clusters in this network and compiled a final library of 4,977 *central motifs*; one motif from each cluster (see Materials and Methods). This extensive motif set displays good coverage of the *Synechocystis* promoters; already at a *q*-value threshold of 0.10 (i.e. less than 10% of the motif mappings are expected to be false positives), virtually every gene had at least one motif mapped and almost every motif in the library was mapped to at least one promoter (Figure 3).

### Motif enrichment in co-expression network neighborhoods of regulatory genes

**Figure 4:**
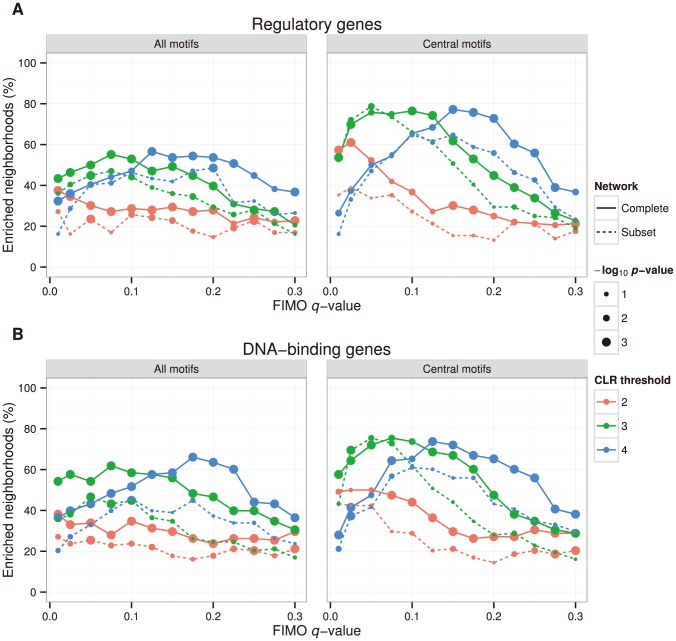
Gene co-expression neighborhoods with significant motif enrichment. The figure plots the fraction of neighborhoods for regulatory genes (A) and DNA-binding genes (B) with at least one significantly enriched motif (*q*<0.05) against the *q*-value threshold for mapping motifs to the genome. The fractions are calculated from the total number of genes in the respective groups that have gene expression data (118 DNA-binding genes and 136 regulatory genes). Plots are shown for all motifs and the subset of central motifs as well as for the complete and subset co-expression networks with different CLR thresholds. *P*-values are given for each combination of parameters and indicate the probability of observing the reported fraction of enriched neighborhoods in randomized networks.

A major aim of our study was to integrate co-expression networks and regulatory motifs in order to describe gene regulation in *Synechocystis*. To this end, we rely on the assumption that genes encoding TFs are co-expressed with their target genes and that the target genes contain a specific binding site, which is used by the TF to initiate transcription. Consequently, we tested this assumption for each gene annotated with a regulatory function or DNA binding by first identifying all genes directly connected to that putative TF (i.e. the *TF neighborhood*) and then by calculating to what degree motifs occurred more often in this neighborhood than what one would expect by chance (i.e. enriched motifs). This analysis was performed for different network CLR thresholds and motif *q*-values in the complete co-expression network and in the subset network (where experiments with missing values were removed) using all discovered motifs and the non-redundant set of central motifs. Figure 4 shows that the library of central motifs resulted in more TF neighborhoods with enriched motifs (*q*<0.05) than the set of all motifs, which on one hand can be explained by the multiple hypothesis correction procedure, but on the other hand also indicates that the reduced set of central motifs covers all motif variants. Also, TF neighborhoods in the complete co-expression network contained enriched motifs more often than in the subset network, indicating that our network inference procedure copes well with data sets having missing values. Based on these results, all analyses are henceforth based on the complete network and the central motifs. Interestingly, there is a relationship between the network CLR threshold and the motif *q*-value threshold, where stricter CLR thresholds require more generous *q*-value thresholds in order to maximize the number of motif-enriched TF neighborhoods. The highest number of enriched TF neighborhoods with the lowest *p*-values was observed in the complete network with a CLR threshold of four and a motif *q*-value of 0.15. Here, 105 of the 136 investigated genes with a regulatory function (77%), and 87 of the 118 investigated DNA binding genes (74%), had at least one enriched motif in its neighborhood. In total, 387 and 445 motifs were enriched in these analyses, respectively. These results are statistically highly significant, both, compared to neighborhoods of ordinary genes in the network (*p* = 0.001) and compared to TF neighborhoods in randomized networks (*p*<0.001). Thus, we can conclude that co-expression and motif information to a large degree concur in *Synechocystis*. The fact that these two completely independent data sets agree so well also strengthens any biological insight inferred from our data.

### Conservation of co-expression in photosynthesis genes

**Figure 5:**
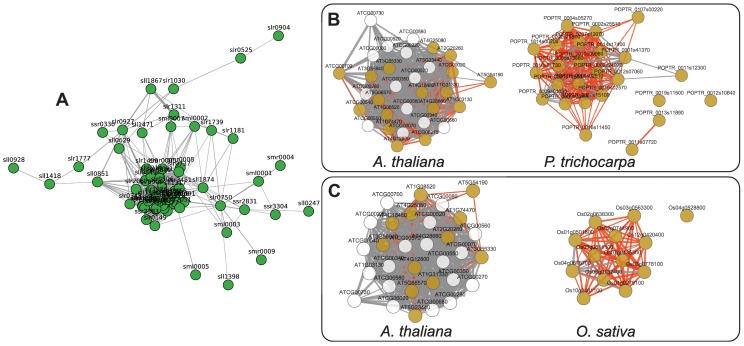
Conservation of photosynthesis genes. Co-expressed genes related to photosynthesis in *Synechocystis* (A) were BLASTed against *A. thaliana*. The orthologs (BLAST E-value <1e-5) were compared against *P. trichocarpa* (B) and *O. sativa* (C) using the network comparison tool ComPlEx. This revealed conservation of co-expression across all four species. Note that the *A. thaliana* genes given in white color were not measurably expressed in the other species.

Cyanobacteria are the evolutionary origin of the plant chloroplast. *Synechocystis* therefore is an important model system for studying photosynthesis. We investigated to what extent the co-expression of *Synechocystis* genes coding for photosynthetic proteins is conserved in plants. 64 *Synechocystis* genes were annotated with the GO term *photosynthesis* (GO:0015979), of which 62 genes formed a connected co-expression subnetwork (CLR threshold of three, Figure 5A). 35 of these *Synechocystis* genes had at least one ortholog in *A. thaliana* (*E*<1e-5), resulting in 30 unique *A. thaliana* gene models (file S3). We analyzed these genes in the comparative network tool ComPlEx [18], and indeed confirmed that all these genes formed a co-expression cluster with the same CLR threshold of three. Moreover, this co-expression network was highly conserved also in *Oryza sativa* and *Populus trichocarpa* (Figure 5B and 5C).

### Web application

We have created a web tool for integrated analysis of co-expression networks and regulatory motifs called *Syn*ergy (http://synergy.plantgenie.org). Available tools include an interactive co-expression network viewer, Gene Ontology and motif enrichment tools, precompiled gene lists and the ability to export annotated gene lists.

**Figure 6:**
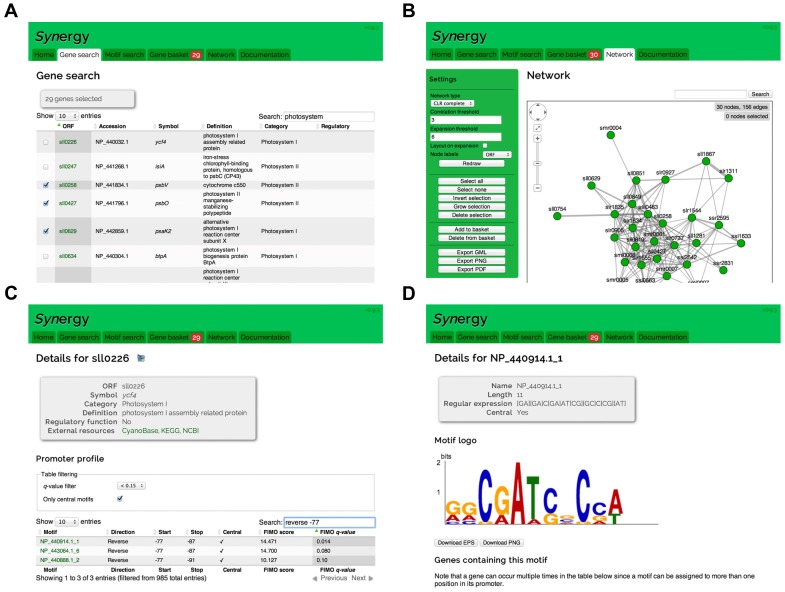
Web application screenshots. Gene search interface (A), network viewer (B), gene details (C) and motif details (D).

The natural starting point on the web site is the *gene search tool*. From here, the user can search for genes of interest or upload a list of genes (Figure 6A). There is also the possibility of using precompiled gene lists; genes annotated to a GO category, genes associated with a motif, genes in a co-expression cluster (Figure 2) and genes in the immediate co-expression neighborhood of a regulatory gene. For each of these gene lists, GO and motif enrichment have been pre-calculated.

Genes of interest can be added to the gene basket and these genes will be available throughout the application. The gene basket page allows the user to manage the gene basket and to calculate GO and motif enrichment for the genes currently in the basket.

The network viewer features the possibility to view and explore co-expression among sets of genes (Figure 6B). Genes that are co-expressed with the gene(s) in the current co-expression network can be found by expanding the network at any selected CLR threshold. It is also possible to export the networks in the Graph Modelling Language (GML) file format, or as publication quality PDFs.

Gene expression profiles of a chosen set of genes can be plotted across the 371 experiments and later downloaded as publication quality PDFs.

For each gene name there is a dedicated page detailing annotations, the expression profile and a list of motifs in the promoter (Figure 6C). Correspondingly, there is a dedicated page for each motif containing the motif logo, the set of genes that contain the motif in their promoters, the possibility of searching for this motif in existing motif databases and the position specific probability matrix for use in other software (Figure 6D).

To make sure that feedback from users reaches the developers by the shortest path possible, a public issue tracker is available at Github (https://github.com/maehler/Synergy/issues). Here, users can file tickets for bugs and enhancements. Documentation for the tools can be found at http://synergy.plantgenie.org/documentation.

Below we describe a number of case studies that illustrate different uses of *Syn*ergy:

### Case study 1: identification of genes regulated by a known transcription factor

**Figure 7:**
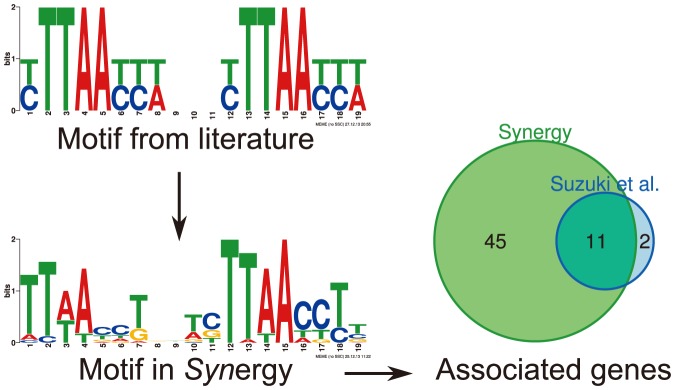
*Syn*ergy case study 1. A regulatory motif and its transcription factor were extracted from the literature [19]. Searching for the motif in *Syn*ergy identified a number of genes that were experimentally determined to be regulated by this transcription factor.

*Syn*ergy can be used to analyze motif occurrences in order to find candidate genes regulated by a known transcription factor. Previously, a spaced motif in the upstream region of genes involved in phosphate limitation had been identified in *Synechocystis* as well as the transcription factor recognizing this motif [19]. The consensus motif contained the direct repeat sequence [CT]TTAA[CT][CT][TA]NNN[CT]TTAA[CT][CT][TA] (Figure 7). Comparing the central region of the motif (TTAA[CT][CT][TA]NNN[CT]TTAA) with existing motifs in *Syn*ergy identified the motif NP_442272.1_1 (*E*-value 1.61e-5). A total of 56 genes contained this motif in their promoter sequence, including *slr0447* (*urtA*), *slr1247* (*pstS2*) and *sll0679* (*sphX*) that have been reported to be up- or down-regulated under phosphate limiting conditions [19]. However, *slr1247* and *sll0679* are leading genes in two operons according to information in Cyanobase. Assuming that the downstream genes in these operons are also regulated by the motif, we identified 11 of the 13 genes reported by [19].

### Case study 2: motif analysis to reveal protein function

*Syn*ergy further can be used to investigate the relationship between a set of genes by integrated analysis of both motifs and co-expression. A search for genes coding for proteins related to the two photosystems in the *Syn*ergy gene search tool resulted in 51 genes that subsequently were tested for regulatory motif enrichment. The motif NP_441569.1_8 was ranked as the second most enriched motif (*q*-value <0.001), and its best match in the Prodoric database was MX000068 in *Bacillus subtilis*. A sigma factor is known to bind to this motif, and using protein BLAST revealed a number of sigma factors with highly significant *E*-values (<1e-10) in *Synechocystis*.

With this information in hand, a new gene search was performed, in which all genes coding for proteins annotated as sigma factors were added to the existing selection of genes. Looking at the co-expression network for these genes revealed that genes coding for photosystems together with those coding for sigma factors formed a connected subnetwork (CLR threshold of three). Our analysis thus supports previous data showing that sigma factors play a vital role in controlling the stoichiometry of the photosystems within the thylakoid membrane [20], [21].

### Case study 3: functional role of hypothetical proteins

*Syn*ergy can be used to assign functions to unknown or hypothetical proteins based on co-expressed genes with known function. The CP12 protein encoded by *ssl3364* is highly conserved in all photosynthetic organisms, but is annotated as a hypothetical protein in Cyanobase. In higher plants and algal species (reviewed by [22]) it was found to be involved in the thioredoxin-mediated regulation of the Calvin-Benson cycle [22]. Moreover, additional functions are hypothesized for this protein in plants [22] and a comparative analysis of 126 cyanobacterial genomes reveals functional diversity among its orthologues [23]. A co-expression neighborhood analysis of *ssl3364* (CLR threshold of four with an expansion threshold of five) generated a densely connected cluster of 54 genes and 798 links. The neighborhood is dominated by genes encoding proteins of the oxidative stress response like chaperones and proteases, and is enriched in genes coding for enzymes involved in protein folding (GO:0006457, *q*-value <0.01). We hypothesize a new biological function for the CP12 protein in *Synechocystis*, *i.e.* protection from oxidative stress, similar to the function of its orthologues in *A. thaliana* and *Chlamydomonas reinhardtii*, which have been shown to protect Calvin-Benson enzymes from oxidative stress [24].

### Case study 4: TF neighborhoods contain biologically relevant motifs

We have shown that the neighborhoods of TFs in our co-expression networks contain common motifs more often than by chance (enriched motifs). To see whether experimental data support that these automatically discovered promoter motifs in fact bind TFs, external motif databases were explored. The gene *sll0998*, for example, encodes a LysR family transcription regulator. In the co-expression network (complete network, CLR threshold of 4) this TF is connected to eight neighboring genes with three enriched motifs in their promoters (*q*<0.05). One of the motifs was NP_440076.1_5. Searching for motifs similar to NP_440076.1_5 in Prodoric resulted in the motif MX000155 known to be regulated by OxyR in *E. coli*. Using protein BLAST to search for homologs of OxyR in *Synechocystis* gave a highly significant hit (*E* = 1e-26) to the protein product of *sll0998*.

## Conclusions

We have developed a web tool, *Syn*ergy, allowing interactive analysis of the *Synechocystis* genome by integrating co-expression networks, regulatory elements and existing knowledge such as functional annotations and known regulatory genes and elements. Furthermore, we have demonstrated the usefulness of this tool in finding both previously published and new biologically relevant regulatory links in *Synechocystis*.

## Materials and Methods

### Microarray data

A total of 371 individual microarray experiments were downloaded from Kyoto Encyclopedia of Genes and Genomes (KEGG; http://www.genome.jp/kegg/expression/). All of the data were based on the Takara microarray chips that covers 83% (3,079/3,726) of the genes in *Synechocystis* [25]. The data were combined into a single data set and normalized with the limma package [26] in R; a software environment for statistical computing and graphics.

### Annotations

Gene annotations were retrieved from Cyanobase. In total, 146 genes were annotated as coding for enzymes with a regulatory function. In this study, these genes were treated as coding for known transcription factors. In Cyanobase, there were also functional annotations translated into GO terms. In total, 2,040 *Synechocystis* genes were annotated to 2,076 GO terms.

### Co-expression inference

Mutual Information (MI) and Context Likelihood of Relatedness (CLR) were used to infer co-expression networks from the microarray data. MI is a metric that does not assume linearity or continuity when measuring the dependence between two variables. This makes it possible to detect relationships that would be undetected by other methods, such as the Pearson correlation coefficient. CLR then finds the most statistically significant co-expression neighbors of each gene based on the local background distribution of MI scores to all other genes [27]. From the z-scores produced by the CLR algorithm, a co-expression network was constructed. A co-expression network can be defined as a collection of nodes (genes) and links (co-expression relationships) where the links are weighted according to the strength of the co-expression.

To account for the large number of missing values in the complete dataset, two different co-expression networks were constructed: the complete co-expression network using all samples (i.e. all 371 microarray experiments) and the subset co-expression network using only the samples with no missing values (67 samples).

### Phylogenetic footprinting

MEME [28] was used to find potential regulatory motifs in groups of orthologs (so-called phylogenetic footprinting). The proteomes of 22 organisms in the Chroococcales taxon (file S2) were downloaded from NCBI and clustered with OrthoMCL [29]. MEME was then used to find conserved motifs in the promoter regions of the corresponding genes in each group. A promoter was defined as the 400 bp sequence upstream of the transcription start site, and the promoters were retrieved using Regulatory Sequence Analysis Tools (RSAT) [30]. MEME was instructed to find motifs between 8 and 20 bp in length with an *E*-value threshold of 100. The MEME motifs were then mapped back to the *Synechocystis* promoters using FIMO [31] and motifs with a *q*-value below 0.3 were kept.

The phylogenetic footprinting approach resulted in many motifs that were similar to each other. To eliminate duplicates, a motif similarity network was constructed. The similarities were calculated by CompariMotif [32] using the consensus motifs derived from the position specific scoring matrices (PSSMs) as input. The motif network was then clustered using MCL [33]. The motif with the highest *betweenness centrality* was chosen as a representative motif from each cluster (central motif).

### Motif and GO enrichment

To calculate enrichment of motifs or GO terms in a set of genes, Fisher's exact test was used. The test was implemented using the Python library scipy (v0.13.3) (http://www.scipy.org). To correct for multiple testing, false discovery rate (FDR) adjustment was used and *q*-values were reported.

### Motif enrichment in network neighborhoods

For genes of interest, the immediate co-expression neighborhood was extracted and motif overrepresentation was calculated for these neighbors. The analysis was performed on genes annotated with *regulatory function* and genes annotated with *DNA-binding*. As a negative control, 1,000 random gene lists with 100 genes in each were used. In all gene sets, genes without expression values were excluded since they will not be present in the co-expression networks. Both, the complete and the subset co-expression networks were used with CLR thresholds of 3, 4 and 5. We also tested different sets of motifs mapped to the genome as defined by different FIMO *q*-value thresholds. For each neighborhood and parameter combination, motif enrichment was calculated using Fisher's exact test and FDR correction as described above, excluding the gene from which the neighborhood was created. If a neighborhood had at least one overrepresented motif with *q*<0.05, the neighborhood was considered to be enriched. To test for significance of the enrichment in the context of networks, motif enrichment was also performed in networks where node labels had been randomly shuffled.

### Web application implementation

The *Syn*ergy web application was developed with the PHP framework CodeIgniter (http://ellislab.com/codeigniter). The network viewer was implemented with the JavaScript library Cytoscape.js (http://cytoscape.github.io/cytoscape.js/), the successor of the Flash interface Cytoscape Web [34].

TOMTOM [35] was used for comparing motifs to known regulatory elements in other organisms. The PRODORIC [36] and RegTransBase [37] prokaryotic motif databases were downloaded from the MEME website.

## Supporting Information

File S1 **GO enrichment of the genes with the highest centrality.**
(XLS)

File S2 **Number of coding regions vs. genome size for the organisms used during the phylogenetic footprinting.**
(XLS)

File S3 **Best sequence alignments with** ***Arabidopsis*** **genes.**
(XLS)
